# Variability of murine bacterial pneumonia models used to evaluate antimicrobial agents

**DOI:** 10.3389/fmicb.2022.988728

**Published:** 2022-09-08

**Authors:** Rakel Arrazuria, Bernhard Kerscher, Karen E. Huber, Jennifer L. Hoover, Carina Vingsbo Lundberg, Jon Ulf Hansen, Sylvie Sordello, Stephane Renard, Vincent Aranzana-Climent, Diarmaid Hughes, Philip Gribbon, Lena E. Friberg, Isabelle Bekeredjian-Ding

**Affiliations:** ^1^Division of Microbiology, Paul-Ehrlich-Institut, Langen, Germany; ^2^Infectious Diseases Research Unit, GlaxoSmithKline Pharmaceuticals, Collegeville, PA, United States; ^3^Department of Bacteria, Parasites & Fungi, Statens Serum Institut, Copenhagen, Denmark; ^4^Infectious Diseases, Evotec, Toulouse, France; ^5^Department of Pharmacy, Uppsala University, Uppsala, Sweden; ^6^Department of Medical Biochemistry and Microbiology, Uppsala University, Uppsala, Sweden; ^7^Fraunhofer Institute for Translational Medicine and Pharmacology ITMP, Discovery Research ScreeningPort, Hamburg, Germany; ^8^Institute of Medical Microbiology, Immunology and Parasitology, University Hospital Bonn, Bonn, Germany

**Keywords:** murine pneumonia model, antimicrobial, lung infection, Gram-negative, PK/PD, antimicrobial efficacy studies

## Abstract

Antimicrobial resistance has become one of the greatest threats to human health, and new antibacterial treatments are urgently needed. As a tool to develop novel therapies, animal models are essential to bridge the gap between preclinical and clinical research. However, despite common usage of *in vivo* models that mimic clinical infection, translational challenges remain high. Standardization of *in vivo* models is deemed necessary to improve the robustness and reproducibility of preclinical studies and thus translational research. The European Innovative Medicines Initiative (IMI)-funded “Collaboration for prevention and treatment of MDR bacterial infections” (COMBINE) consortium, aims to develop a standardized, quality-controlled murine pneumonia model for preclinical efficacy testing of novel anti-infective candidates and to improve tools for the translation of preclinical data to the clinic. In this review of murine pneumonia model data published in the last 10 years, we present our findings of considerable variability in the protocols employed for testing the efficacy of antimicrobial compounds using this *in vivo* model. Based on specific inclusion criteria, fifty-three studies focusing on antimicrobial assessment against *Pseudomonas aeruginosa, Klebsiella pneumoniae* and *Acinetobacter baumannii* were reviewed in detail. The data revealed marked differences in the experimental design of the murine pneumonia models employed in the literature. Notably, several differences were observed in variables that are expected to impact the obtained results, such as the immune status of the animals, the age, infection route and sample processing, highlighting the necessity of a standardized model.

## Introduction

Antimicrobial resistance is recognized as one of the greatest threats to human health (World Health Organization [WHO], 2017; Morehead and Scarbrough, 2018; Murray et al., 2022). Thus, new antimicrobial therapies are urgently needed, although few are currently being developed (Hughes and Karlén, 2014; Bekeredjian-Ding, 2020; Theuretzbacher et al., 2020). Due to numerous challenges, including long research timelines and limited financial reward, most large pharmaceutical companies are no longer investing in research and development of new antibiotics. To ensure a sustainable pipeline of novel therapies, improving the efficiency and attractiveness of antibiotic drug development is crucial.

Animal models are essential to bridge the translational gap between preclinical and clinical research (Denayer et al., 2014; Friberg, 2021). They provide an infection environment and anatomical barriers that are difficult to reproduce *in vitro*, and they can be very useful in predicting potentially efficacious dosing regimens (Bulitta et al., 2019; Tängdén et al., 2020). Several different mammalian species have been used to model human pneumonia including piglets (Li Bassi et al., 2014), rodents (Mizgerd and Skerrett, 2008), non-human primates (Kraft et al., 2014), sheep (Malachowa et al., 2019), and rabbits (Nguyen et al., 2021). Although these models have proven helpful in studies of disease mechanisms and in antibiotic testing, murine models have been the preferred choice in investigational new drug applications for the treatment of bacterial pneumonia (Waack et al., 2020). Despite anatomical and physiological differences, the immune system of mice mimics that of humans and pathology of murine pneumonia resemble features of human pneumonia (Mizgerd and Skerrett, 2008; Metersky and Waterer, 2020). However, the observed pathology in mice strongly depends on pathogen-specific features of virulence, route of infection, infectious dose and other factors such as animal genetic background (Mizgerd and Skerrett, 2008; Bielen et al., 2017; Dietert et al., 2017). The features and measurements of experimental acute lung injury in animals depend on the experimental question to be addressed and it has been discussed elsewhere (Matute-Bello et al., 2011). The advantages of using murine models include ease of handling and cost effectiveness. Standardization of the mouse pneumonia model is deemed necessary to improve the robustness and reproducibility in preclinical studies and therefore improve translational research (Peers et al., 2012; Begley and Ioannidis, 2015). In order to improve the reproducibility of results and to facilitate comparisons between studies, it is important to report any data that could potentially influence the outcome. Despite the development of specific guidelines such as TOP (Transparency and Openness Promotion; Nosek et al., 2015), ARRIVE (Animal Research: Reporting of In Vivo Experiments; Kilkenny et al., 2010; Percie du Sert et al., 2020) or PREPARE (Planning Research and Experimental Procedures on Animals: Recommendations for Excellence; Smith et al., 2018), there are still considerable gaps and discrepancies in the experimental information reported in the scientific literature. Establishing a standard method that includes key information can help researchers to navigate through essential variables and ensure that described study protocols are both complete and adequately detailed as well as reported in a consistent and standardized manner. The use of standardized animal model avoids the time-consuming process of developing *in vivo* protocols and reduces the variability of the results. Therefore, it adheres to the 3R principle, reducing the number of animals required in preclinical studies. In addition, the development of a standardized murine pneumonia model validated with at least one reference compound will enable antibiotic benchmarking and serve as a quality control mechanism of the results obtained between laboratories.

The European Innovative Medicines Initiative (IMI) Antimicrobial Resistance (AMR) Accelerator was created with the main goal of advancing the development of new medicines to treat or prevent resistant bacterial infections worldwide. Within the AMR Accelerator, the “Collaboration for prevention and treatment of MDR bacterial infections” (COMBINE) consortium aims to develop a standardized, quality-controlled murine pneumonia model for preclinical efficacy testing of novel anti-infective candidates and to improve tools for the translation of preclinical data to the clinic. Success in translational medicine heavily depends on the selected animal models and the experimental set up of the animal model (Hooijmans and Ritskes-Hoitinga, 2013; Denayer et al., 2014). In addition, the success of characterizing pharmacokinetics and pharmacodynamic (PK/PD) targets in animal models relies largely on host and microbial study design features and the ability to control variance (Andes and Craig, 2002; Andes and Lepak, 2017; Bulitta et al., 2019). Although recommendations for *in vivo* PK/PD studies have been published (Andes and Lepak, 2017; Bulitta et al., 2019), there is still a need for globally harmonized preclinical models.

This focused literature review aims to describe the variability in study methods and experimental protocols for the mouse lung infection model used to test antimicrobial efficacy. This is an essential preliminary step to advance the development of standardized preclinical animal models. Our review focused on murine lung infection models of the most relevant MDR Gram-negative pathogens, *Pseudomonas aeruginosa, Klebsiella pneumoniae* and *Acinetobacter baumannii*, used in proof-of-concept and/or primary pharmacology studies for small molecule antibiotics. The findings were further shared and discussed by a panel of experts at an online workshop organized by the COMBINE consortium. The resulting recommendations for standard design parameters are presented in the following joint article: “Expert Workshop Summary: Advancing toward a standardized murine model to evaluate treatments for AMR lung infections” and they will provide the basis for the development of a harmonized and bench-marked murine lung PK/PD model.

## Literature search strategy, study selection and publication characteristics

Established protocols for murine pneumonia models were collected from industrial, academic, and governmental institutions. A total of sixteen protocols from ten different institutions were reviewed and compared to create a list of parameters that varied between protocols. Furthermore, a scientific literature search was performed to investigate the variability of mouse pneumonia model protocols in published studies. Parameters from the established institutional protocols were excluded from the data analysis of the literature findings.

**FIGURE 1:**
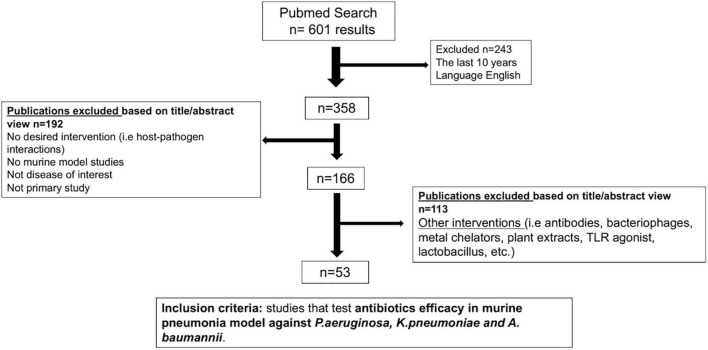
Flow diagram for the study selection process.

Study selection followed SYstematic Review Center for Laboratory animal Experimentation (SYRCLE) guidelines (Leenaars et al., 2012). The search strategy consisted of the identification and definition of three search components: mouse model, pneumonia caused by *P. aeruginosa, K. pneumoniae* and/or *A. baumannii*, and drug therapy. A total of 25 Mesh terms and 13 free text terms limited to the title and abstract were used for a literature search in PubMed (Supplementary Table 1). The studies selection process is summarized in Figure 1. A total of 601 preliminary studies were retrieved, of which 358 studies were published within the last decade in the English language. Of these, 192 publications were excluded following a title and abstract review due to not being primary studies, the disease of interest (murine pneumonia model), or not being focused on the desired intervention; thus, 166 publications were considered to be initially relevant. Following the exclusion of additional studies that focused on interventions or therapies other than small molecule antibiotics (monoclonal antibodies, bacteriophages, metal chelators, plant extracts, *Lactobacillus*, etc.), 53 studies remained (López-Rojas et al., 2011; Pachón-Ibáñez et al., 2011; Docobo-Pérez et al., 2012; Tang et al., 2012; Wang et al., 2012; Yamada et al., 2012, 2013a,b; He et al., 2013; Hirsch et al., 2013; Jacqueline et al., 2013; Louie et al., 2013, 2015; Harada et al., 2014; Hengzhuang et al., 2014; Yokoyama et al., 2014; Bowers et al., 2015; Cheah et al., 2015; Berkhout et al., 2016; Brunetti et al., 2016; Cigana et al., 2016; Lepak and Andes, 2016; Mardirossian et al., 2016; McCaughey et al., 2016; Parra Millán et al., 2016; Thabit et al., 2016; Yang et al., 2016; Kaku et al., 2017a,b; Li Y. T. et al., 2017, Li Y. et al., 2017; Lin et al., 2017a,b, 2018; Oshima et al., 2017; Sakoulas et al., 2017; Zhou J. et al., 2017, Zhou Y. F. et al., 2017; Avery et al., 2018; Chen et al., 2018; de Paula et al., 2018; Geller et al., 2018; Lou et al., 2018; Monogue et al., 2018; Kirby et al., 2019; Ku et al., 2019; Nakamura et al., 2019; Ren et al., 2019; Sanderink et al., 2019; Zhao et al., 2019; Johnson et al., 2020; Tan et al., 2020; Ma X. L. et al., 2020; Supplementary Table 2). Murine model variables were extracted from these articles to generate a data set for further analyses of experimental conditions. The data set contained studies from 14 different countries published from 2011 to 2020 (Supplementary Table 2).

## Review of key variables in murine bacterial pneumonia models used to evaluate antimicrobial agents

### Bacterial and intervention-related variables

**FIGURE 2:**
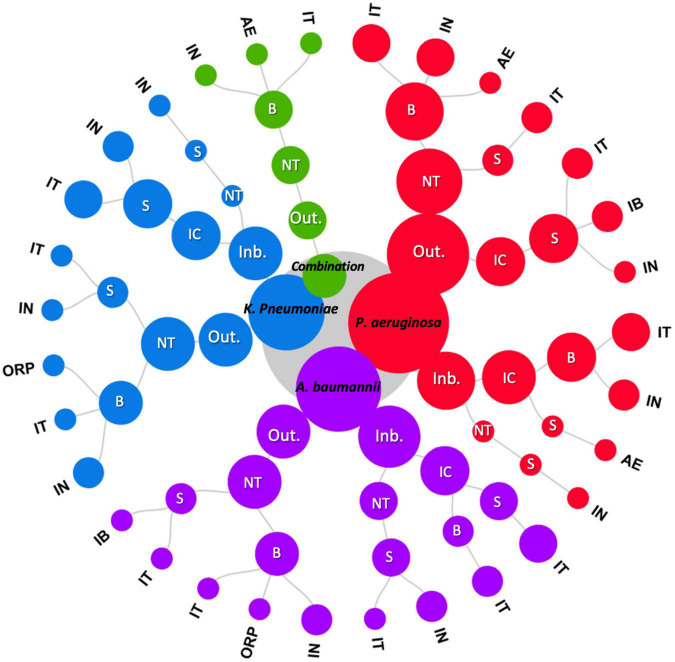
Circular dendrogram representing hierarchically structured variables. The area of the nodes represents the number of studies. Hierarchy from inside to outside: bacteria, mouse strain (Inb.: Inbred, Out.: Outbred), mouse immune status (NT: neutropenic, IC: Immunocompetent), study main readout (S: mice survival, B: bacterial load) and infection route (IT: intratracheal, IN: intranasal, ORP: oropharyngeal, IB: intrabronchial, AE: aerosolization).

*Pseudomonas aeruginosa*, *Klebsiella pneumoniae* and *Acinetobacter baumannii* are among the most common and difficult to treat opportunistic pathogens in nosocomial infections such as ventilator-associated pneumonia in immunocompromised patients (Ma Y. et al., 2020). We observed that antibiotic efficacy was most commonly evaluated against *P. aeruginosa* with 22 of the 53 studies reviewed including this pathogen. This pathogen is commonly involved in pneumonia of cystic fibrosis patients (Oliver et al., 2000). Although antimicrobial agents may be efficacious against more than one of these Gram-negative pathogens (Paterson et al., 2020), only a few of the published studies included the *in vivo* efficacy against two or three of the pathogens in separate experiments (termed “combination of bacteria,” Table 1 and Figure 2). Evaluation of antibacterial monotherapy was the most common study objective in the studies reviewed, although studies with *A. baumannii* focused mostly on the evaluation of a combination of therapies (18.9% of studies). Despite the increased attention given to drug delivery methods (Li et al., 2019), few studies have focused on the evaluation of alternative routes of drug administration (Table 1). This consisted mainly of aerosolization or liposomes for pulmonary administration of colistin and polymyxin B (He et al., 2013; Li Y. et al., 2017; Lin et al., 2017a,b, 2018). *P. aeruginosa* was the pathogen of choice for these investigations (7.5% of all studies, Table 1).

**TABLE 1:** Bacterial and intervention variables in the reviewed studies.

		Number of studies	Percentage (%)
**Bacteria**			
*P. aeruginosa*		22	41.5
*K. pneumoniae*		12	22.6
*A. baumannii*		15	28.3
Combination of bacteria		4	7.5
**Main objective of the study**			
Evaluation of drug monotherapy		25	47.2
	*P. aeruginosa*	12	22.6
	*K. pneumoniae*	6	11.3
	*A. baumannii*	4	7.5
	Combination of bacteria	3	5.7
Evaluation of drug combination therapy		22	41.5
	*P. aeruginosa*	6	11.3
	*K. pneumoniae*	6	11.3
	*A. baumannii*	10	18.9
	Combination of bacteria	0	0.0
Evaluation of alternative drug delivery		6	11.3
	*P. aeruginosa*	4	7.5
	*K. pneumoniae*	0	0.0
	*A. baumannii*	1	1.9
	Combination of bacteria	1	1.9
**Number of strains**			
1 or 2 strains		38	71.7
	*P. aeruginosa*	19	35.8
	*K. pneumoniae*	8	15.1
	*A. baumannii*	11	20.8
	Combination of bacteria	0	0.0
3 or 4 strains		11	20.8
	*P. aeruginosa*	3	5.7
	*K. pneumoniae*	3	5.7
	*A. baumannii*	2	3.8
	Combination of bacteria	3	5.7
More than 5 strains		4	7.5
	*P. aeruginosa*	0	0.0
	*K. pneumoniae*	1	1.9
	*A. baumannii*	2	3.8
	Combination of bacteria	1	1.9
**Bacterial source**			
Own clinical isolate		25	47.2
	*P. aeruginosa*	11	20.8
	*K. pneumoniae*	4	7.5
	*A. baumannii*	10	18.9
Collaborator		5	9.4
	*P. aeruginosa*	3	5.7
	*K. pneumoniae*	1	1.9
	*A. baumannii*	1	1.9
Strains Bank		6	11.3
	*P. aeruginosa*	1	1.9
	*K. pneumoniae*	4	7.5
	*A. baumannii*	1	1.9
Not reported		17	32.1
**Bacteria growth stage**			
Frozen log. phase stock		2	3.8
Subcultured to log. phase		13	24.5
	Early log. phase	4	7.5
	Mid-log. phase	1	1.9
	Not reported	8	15.1
Not reported		38	71.7

Percentages over 53 total studies reviewed.

There are no firm requirements for the number of bacterial strains to be included in preclinical studies for the evaluation of novel antimicrobials *in vivo.* However, regulatory guidance (European Medicines Agency, 2016) and scientific recommendations (Bulik et al., 2017; Bulitta et al., 2019) suggest to include at least four strains of each target pathogen species for establishing PK/PD targets. Ideally, these should include a reference strain and be representative of contemporary, relevant resistance profiles and mechanisms (European Medicines Agency, 2016; Bulik et al., 2017; Bulitta et al., 2019). We observed that most of the studies included only one or two bacterial strains, especially those focusing on *P. aeruginosa* (35.8% of all studies). Less than one third of the studies tested three or more strains of the same species (Table 1). The value of testing several strains is that it accounts for genetic and biological variation within the target species which may affect strain fitness and susceptibility and therefore the overall efficacy assessment of the investigated drug (Andes and Lepak, 2017).

The source of the bacterial strain was reported in 67.9% of reviewed studies (Table 1). Private clinical isolates were the most common source of bacteria for *A. baumannii* and *P. aeruginosa*. The lack of globally accessible reference strains with corresponding *in vivo* benchmark data may partially explain why some studies used a strain obtained from another researcher (Table 1). Therefore, it would be highly recommended to deposit *in vivo* pathogenic strains (and associated data) in biorepositories to make them accessible to other researchers.

Another variable expected to impact bacterial fitness and infectivity is the preparation of the bacterial inoculum. However, the details related to the inoculum preparation procedure were rarely included. Only 28.3% of studies reported the bacterial growth stage at the time of infection. Of these, a fresh bacterial subculture in logarithmic phase of growth was generally employed for the infection and the use of frozen stocks was limited (Table 1). Some virulence factors are differentially expressed between logarithmic and stationary phase. Their expression often increases in stationary phase, when the cell density is high and bacteria are subjected to higher biological stress (Carter et al., 2007; Bravo et al., 2008; Choi et al., 2011). *P. aeruginosa* quorum sensing signal increases at late stationary phase (Choi et al., 2011). In *Salmonella* and *Shigella*, the production of the lipopolysaccharide long-chain O-antigen increases in the late exponential and stationary growth phases, which affects serum resistance (Carter et al., 2007; Bravo et al., 2008). Despite this, bacteria in logarithmic phase are overwhelmingly employed for *in vivo* infection for a number of reasons. First, the ability of logarithmic phase bacteria to survive and establish a robust infection in the lung is consistently reproducible. Second, logarithmic phase bacteria can be more accurately quantified in the inoculum. It is technically challenging if not impossible to produce a stationary phase culture containing no dead cells. The effects, of employing bacterial cultures in different growth stages or bacteria cultured on liquid vs solid media is currently unknown. Bacterial growth stage is rarely reported; and very few studies reported the specific stage of the log phase (Table 1). However, to be able to increase the reproducibility of the preclinical studies, it is recommended to describe the culture conditions precisely and occasionally to monitor the expression of virulence factors.

### Animal-related variables

The selection of mouse strain is a choice that should be carefully considered. Outbred mice were used more frequently than inbred mice. Swiss Webster mice were the most common outbred stock, followed by ICR mice. With regard to inbred mice, C57BL/6 and BALB/c mice were used most frequently (Table 2). The preference for inbred vs. outbred mice varied depending on the bacterial species under investigation. Studies performed with *P. aeruginosa* mainly used outbred mice, while for *A. baumannii* inbred mice slightly predominated (Figure 2). When working with *K. pneumoniae*, inbred and outbred mice were used to a similar extent (Figure 2). Outbred mice are generally selected for PK/PD studies in murine lung and thigh models (Andes and Lepak, 2017; Bulitta et al., 2019). It has been described that *Pseudomonas* infection led to higher mortality in BALB/c mice (classified as a Th2 responder strain) compared to C3H/HeN mice (Th1 responders; Moser et al., 1997) and C57BL/6 mice are more susceptible to *Klebsiella* infection of the lung than are 129/Sv mice (Schurr et al., 2005). Inbred mice are presumed to be more uniform (thus decreasing the number of animals needed to detect a specific response) and more repeatable (a result of being genetically defined and less prone to genetic change; Festing, 2014). However, to date there is no evidence of greater trait stability in inbred mice. This suggests that the advantages of inbred mice may not be as great as previously supposed and that the use of outbred mice in biomedical research may provide an important advantage in reaching conclusions that are generalizable across conditions and populations (Tuttle et al., 2018).

**TABLE 2:** Characteristics of mice in the reviewed studies.

		Number of studies	Percentage (%)
**Mouse strain**			
Inbred		22	41.5
	C3H/HeN	1	1.9
	BALB/c	8	15.1
	C57BL/6	12	22.6
	DBA/2	1	1.9
Outbred		29	54.7
	NMRI	2	3.8
	Swiss Webster	12	22.6
	CD-1	3	5.7
	ddY	5	9.4
	ICR	6	11.3
	Kunming	1	1.9
Not reported		2	3.8
**Sex**			
Female		34	64.2
Male		9	17.0
Female and male		1	1.9
Not reported		9	17.0
**Age average**			
**Inbred**			
	4–5 weeks	0	0.0
	6–7 weeks	11	21.6
	≥8 weeks	10	19.6
	Not reported	1	2.0
**Outbred**			
	4–5 weeks	6	11.8
	6–7 weeks	15	29.4
	≥8 weeks	6	11.8
	Not reported	2	3.9
**Number of animals per group**			
**Bacterial load^1^**			
	2–3	12	23.5
	4–6	27	52.9
	7–10	9	17.6
	11–15	3	5.9
**Survival^2^**			
	2–3	0	0.0
	4–6	5	20.8
	7–10	12	50.0
	11–15	7	29.2

^1^Number of animals per group employed when bacterial load was the main readout.

^2^Number of animals per group employed when survival was the main readout.

The sex of experimental animals is known to impact host-pathogen interactions (García-Gómez et al., 2013). We observed that the vast majority of the studies were carried out in female mice regardless of the bacteria used for infection (Table 2). Interestingly, only one study used both female and male mice in separate experiments with different readouts (Kirby et al., 2019; Table 2). Female C57BL/6J mice have been shown to be more susceptible to *A. baumannii* lung infection than their male counterparts (Pires et al., 2020). However, an increased susceptibility of male C3HeB/FeJ mice has been reported in the oral aspiration pneumonia model (Luna et al., 2019). In humans, women with cystic fibrosis and *P. aeruginosa* infection have worse outcomes than men (Demko et al., 1995), which has been partly attributed to estrogen effects (Vidaillac et al., 2020). In the case of *K. pneumoniae* infection, female mice have showed higher survival rates than males although exposure to ozone reversed the trend and resulted in female mice surviving less than males (Mikerov et al., 2011). The sex of the test animal can also have implications in drug PK/PD (Soldin and Mattison, 2009; Madla et al., 2021). Therefore, the selection of male or female mice in an antimicrobial efficacy study is a variable that should be carefully considered and taken into account when interpreting the results.

The age of mice used in the study could substantially affect the immune response and thus the infection outcome (Cai et al., 2016; Jackson et al., 2017). Older mice have a more mature immune response. It has been shown in mice that B-cells have an immature phenotype until 4 weeks of age (Ghia et al., 1998) and T-cell responses mature around 8 weeks of age (Holladay and Smialowicz, 2000). In addition, drug metabolism by the liver is affected by the age of mice and has a critical impact on systemically administered compounds (Pibiri et al., 2015). This variable is also related to the weight of the animal and in some cases this parameter is reported instead of the age. For data analysis, we transformed the weight of the mice (if it was the only data provided) to age in weeks according to the vendor’s growth data provided for the mouse strain of interest. The age of mice ranged from 4 to 10 weeks and animals with an average age of 6 weeks were most common. Outbred mice tended to be younger than inbred mice, and no inbred mice younger than 6 weeks were used (Table 2). Although outbred mice grow faster than inbred mice, their immune system may not be fully developed at the selected age (Ghia et al., 1998; Holladay and Smialowicz, 2000). This may be less of a problem for studies using outbred animals, as they are often rendered neutropenic or used at older ages in immunocompetent models. Several guidelines such as ARRIVE have strongly encouraged reporting the age of animals used in an experiment (Kilkenny et al., 2010; Percie du Sert et al., 2020). However, it is not clear if the age reported in the studies refers to the age of an animal upon arrival at the facility (before the acclimatization period), the age when the experimental infection is performed or the age when the first intervention in the animals is executed (i.e., cyclophosphamide treatment). This could lead to even greater variation in age than immediately apparent. The host microbiota correlates with animal age and affects host response and lung infection resistance (McMahan et al., 2022). The mouse microbiome varies based on a number of factors, including the animal’s origin, nutrition, housing, bedding, and care during infancy (Ericsson and Franklin, 2021). Around 6 weeks of age, the lung’s microbial diversity significantly increases and is maintained throughout time (Singh et al., 2017). Researchers should make an effort to minimize the effects of this factor on the host’s susceptibility to infection in order to improve the reproducibility of the studies.

The number of animals per group should be selected based on a power analysis considering expected data dispersion (Festing, 2018; Bulitta et al., 2019). We observed broad variation in the number of animals included per group. In studies with bacterial burden as the main endpoint, a range of two to fifteen animals was used, with four to six animals per group in most of the studies. In studies with survival as the study endpoint, the most common group size was 7–10 animals per group, reflecting that survival data typically present higher variability than bacterial burden (Table 2).

### Infection procedure-related variables

Induced neutropenia is a variable that can have a significant impact on the study outcomes (Andes and Craig, 2002; Andes and Lepak, 2017). In general, a higher PK/PD index magnitude is required in neutropenic animals although it varies among drug classes and bacterial species (Andes and Craig, 2002; Andes and Lepak, 2017). We observed that neutropenic mice were used more often than immunocompetent mice (Table 3). Immunocompetent mice were preferred when working with inbred mouse strains, while the use of neutropenic animals prevails when outbred mice were used. This confirms the results of a previous literature review that evaluated different animal models for antibiotic efficacy testing, which found that neutropenic models slightly predominate over immunocompetent ones (Waack et al., 2020). Our analysis showed that the immune status of the mice varied by the study outcome measured. Survival was most frequently evaluated in immunocompetent animals. *P. aeruginosa* studies employed mostly immunocompetent lung models whereas *A. baumannii* and *K. pneumoniae* infections were mostly conducted in neutropenic models (Figure 2). Neutropenic animals were found to have increased bacterial growth over the study period in untreated animals (Table 4). Despite a common misconception, use of immunocompromised mice is not generally intended to mimic any particular patient population (Zhao et al., 2016; Andes and Lepak, 2017). Neutropenia promotes better growth of bacteria in mice, thus minimizing spontaneous resolution of infection (which complicates interpretation of treatment effects) and enabling more strains to be studied in mice than otherwise might be possible.

**TABLE 3:** Inoculation and procedural parameters in the reviewed studies.

		Number of studies	Percentage (%)
**Immune status**			
*Inbred*		22	43.1
	Immnunocompetent	16	31.4
	Neutropenic	5	9.8
	Immnunocompetent and neutropenic	1	2.0
*Outbred*		29	56.9
	Immnunocompetent	5	9.8
	Neutropenic	24	47.1
**Infectious route**			
*Immunocompetent*		22	42.3
	Aerosolization	1	1.9
	Intrabronchial	2	3.8
	Intranasal	5	9.6
	Intratracheal	14	26.9
*Neutropenic*		30	57.7
	Aerosolization	2	3.8
	Intrabronchial	1	1.9
	Intranasal	14	26.9
	Intratracheal	11	21.2
	Oropharyngeal	2	3.8
**Infectious volume**			
	10 μl	2	4.3
	20 μl	4	8.5
	25 μl	9	19.1
	30 μl	4	8.5
	40 μl	3	6.4
	50 μl	23	48.9
	70 μl	2	4.3
**Infectious dose**			
<5 log_10_ CFU		4	8.3
	*P. aeruginosa*	3	6.3
	*K. pneumoniae*	1	2.1
	*A. baumannii*	0	0.0
5–6 log_10_ CFU		8	16.7
	*P. aeruginosa*	2	4.2
	*K. pneumoniae*	2	4.2
	*A. baumannii*	4	8.3
6–7 log_10_ CFU		16	33.3
	*P. aeruginosa*	9	18.8
	*K. pneumoniae*	4	8.3
	*A. baumannii*	3	6.3
7–8 log_10_ CFU		19	39.6
	*P. aeruginosa*	7	14.6
	*K. pneumoniae*	5	10.4
	*A. baumannii*	7	14.6
>8 log_10_ CFU		1	2.1
	*P. aeruginosa*	0	0.0
	*K. pneumoniae*	0	0.0
	*A. baumannii*	1	2.1

**TABLE 4:** Treatment and procedural parameters in the reviewed studies.

		Number of studies	Percentage (%)
**Treatment start point (p.i.)**			
	≤1 h	8	15.1
	2 h	22	41.5
	3–4 h	12	22.6
	5–6 h	3	5.7
	>6 h	8	15.1
**Experimental endpoint (p.i.)**			
***Bacterial load* ^1^**			
	12–18 h	2	3.8
	24–26 h	25	48.1
	27–30 h	5	9.6
	>36 h	7	13.5
Several time points (between 2 and 72 h)		13	25.0
***Survival* ^2^**			
	≤2 d	3	10.7
	3–4 d	12	42.9
	5–6 d	5	17.9
	7–9 d	6	21.4
	10–11 d	2	7.1
**Baseline bacterial burden (CFU per lung)**			
	4.7–6	1	1.89
	6–7	9	16.98
	7–8	5	9.4
	Not reported	38	71.7
**Average bacterial growth in lung (CFU per lung)**			
***Neutropenic mice***			
	≤1	2	5.9
	1–2	7	20.6
	2–3	9	26.5
	>3	5	14.7
***Immunocompetent mice***			
	≤1	6	17.6
	1–2	2	5.9
	2–3	3	8.8
	>3	0	0.0
**Study readouts**			
Survival		26	49.1
Bacterial load		50	94.3
Blood or spleen or liver CFU		11	20.8
Histopathology		16	30.2
Immune response		11	20.8
Others^3^		5	9.4

^1^Experimental endpoint in hours (h) when bacterial load was the main readout.

^2^Experimental endpoint in days (d) when survival was the main readout.

^3^Other readouts measured: clinical score, body temperature, protein expression and lung endothelial permeability.

Neutropenia can be induced by different methods, including the use of drugs such as cyclophosphamide or vinblastine and neutrophil depleting antibodies (Stackowicz et al., 2020). Cyclophosphamide has a relative low cost and produce depletion of hematopoietic stem cells associated with an almost complete disappearance of blood neutrophils as early as 3–4 days after injection (Van’t Wout et al., 1989; Zuluaga et al., 2006). Cyclophosphamide has the greatest effect on neutrophil numbers, but also markedly reduces numbers of circulating monocytes, B and T cells (Van’t Wout et al., 1989; Zuluaga et al., 2006). Cyclophosphamide was the only methodology employed in the reviewed studies to render animals neutropenic. More than half of the studies with immunocompromised mice used a protocol of administering cyclophosphamide 4 days (150 mg/kg) and one day (100 mg/kg) before infection. The remaining studies used slight variations, such as increasing the administered dose or varying the days of administration.

The route of infection has been shown to impact host-pathogen interactions (Martins et al., 2013). In the reviewed studies, intratracheal (IT) and intranasal (IN) routes of infection were the most common, followed by aerosolization, the intrabronchial route and oropharyngeal infection route. Infections with *A. baumannii* and *P. aeruginosa* were mostly achieved through IT bacterial inoculation, while *K. pneumoniae* infection protocols mostly used IN bacterial administration (Figure 2). Moreover, we observed that the selection of the infection route was related to the immune status of the animal. IN infection was mostly employed when working with neutropenic animals, while for immunocompetent animals, IT infection was most common (Table 3). The predominance of the IN route when using neutropenic animals and the IT when employing immunocompetent animals was also observed in a previous report that summarized studies of antibiotic efficacy against seven gram-negative and two gram-positive pneumonia-causing bacterial species (Waack et al., 2020). This variation may reflect the challenge of establishing lung infections in immunocompetent mice, where a more direct inoculation method such as IT route can lead to a greater success. In our reviewed studies using three gram-negative bacteria, we observed a higher percentage of immunocompetent animals infected *via* IT route than previously reported using a higher number of pneumonia-producing bacteria (17), suggesting that the infection with these three selected gram-negative bacteria may be more difficult to achieve.

The infection volume is closely related to both the employed route of infection and the infectious dose. The administered volume ranged from 10 to 70 μl and most of the studies used a volume of 50 μl (Table 3). The lowest volume of 10 μl was only used for IT bacterial delivery (He et al., 2013; Hirsch et al., 2013).

It has previously been shown in a murine model of tularemia that intranasal instillation of a volume of 10 μl routinely resulted in infection of the upper airways but failed to initiate infection of the pulmonary compartment. For efficient delivery of the bacteria into the lungs, a dose volume of 50 μl or more was required (Miller et al., 2012). Similarly, studies with the azo dye Evans blue have revealed that intranasal administration of a dye volume of 40–50 μl, in comparison to 10–20 μl, led to increased dye retention in the lungs (Visweswaraiah et al., 2002; Smith et al., 2019). These data suggest that infecting volumes of at least 50 μl IN are preferred for establishing robust pulmonary infection.

Before selecting bacterial strains and infectious dose to perform antimicrobial efficacy studies, bacterial pathogenicity studies in mice are required. The infectious inoculum varied greatly between the reviewed studies. Most indicated that 7–8 log_10_ CFU were administered to initiate the infection (Table 3). Studies working with *P. aeruginosa* and *K. pneumoniae* used lower infectious dose than the studies working with *A. baumannii* (Table 3). Considering that the neutropenic model already prevails when working with this pathogen (Figure 2), these data may suggest greater difficulty in establishing a robust *A. baumannii* lung infection, requiring higher infectious dose.

### Treatment and readout-related variables

In antibacterial research, the length of the study varies between animal species. Murine pneumonia models are mostly used for short term studies, while larger animals usually employ later endpoints (Waack et al., 2020). The vast majority of the studies reviewed here initiated antibiotic therapy at 2 h post infection (h.p.i.). However, the timing of the experimental endpoint varied according to the main study readout. Studies to determine bacterial burden were mostly terminated at 24 or 26 h.p.i., or at multiple time points between 2 and 72 h.p.i. Survival studies were most commonly terminated after 3–4 days (Table 4). A previous review of new drug application dossiers found that an endpoint of 24–29 h.p.i. was most commonly employed when antibacterial activity against *P. aeruginosa*, *K. pneumoniae* and *A. baumannii* was investigated. Notably, these studies followed a bimodal distribution, having 24- or 48-h endpoints (Waack et al., 2020). The endpoint used for measuring antimicrobial activity could influence the results (Andes and Craig, 2002; Cigana et al., 2020); however, the evaluation of antimicrobial treatments for chronic infection requires longer experimental time points (Cigana et al., 2020). Additionally, there might be variations in how researchers analyze animal care and humane endpoints in survival studies. Clinical disease severity scoring can be subjective, with various researchers scoring severity differently.

Unfortunately, the lung bacterial burden at the start of therapy was only reported in 28.3% of the reviewed studies. Baseline burdens fluctuated from 4.7 to 8 log_10_ CFU per lung, with the majority falling into a range of 6–7 log_10_ CFU per lung (Table 4). A lower burden at the start of therapy may reduce the PK/PD index magnitude, thus reducing the dose required for treatment effect, although the degree of influence can vary depending on the bacterial species and the drug class (Andes and Lepak, 2017). Importantly, the majority of results from PK/PD animal models that have been correlated with clinical outcomes reached 6–7 log_10_ CFU in the target tissue at the time therapy was initiated (Andes and Lepak, 2017). Baseline burdens further affect the bacterial growth over the study period. Bacterial growth was reported in 34 of the 53 studies reviewed, and an increase of at least 1 log_10_ CFU/lung over the experimental period (ranged from 2 and 72 h.p.i, median 26 h.p.i) was achieved in most of the studies. There were no marked differences among the three different bacterial species. However, bacterial growth over the study period was indeed related to immune status, with neutropenic mice showing higher bacterial growth than immunocompetent mice (Table 4). Robust infections are a prerequisite to adequately assess the antimicrobial effect of drugs. Therefore, bacterial burdens in untreated animals should not decline over the course of the study, or it becomes difficult to separate treatment effect from spontaneous resolution of infection. Depending on the selected efficacy endpoint, the effect of treatment may also be overestimated if bacterial growth is poor. The recommended specific efficacy endpoints range from 0 (stasis) to 2 log_10_ reductions in CFU calculated relative to the bacterial density at the start of treatment (Drusano, 2004; European Medicines Agency, 2016; Bulitta et al., 2019) and there remains some debate over which should be used. Stasis may be adequate for less severe infections or those involving concomitant non-drug treatments (e.g., surgical intervention such as debridement of infected tissue). For infections involving skin, soft tissue or the urinary tract, a 1 log_10_ reduction has been recommended, whereas 2 log_10_ reductions have been recommended for severe and/or high bacterial burden infections such as pneumonia (Drusano et al., 2018; Bulitta et al., 2019). In the control group (untreated or vehicle-treated), an increase in bacterial burden of 2–3 log_10_ CFU over the course of the study has been recommended (Bulitta et al., 2019). While this may be a feasible target for the standard neutropenic thigh infection model, studies reviewed suggest that it may be more difficult to achieve in the lung model especially with some pathogens (e.g., *A. baumannii*) or bacterial strains.

In the studies reviewed, specific information on sample processing was scarce. The method of lung homogenization was only reported in 15% of the studies. Stomacher, mini bead beater and ultra-turrax were the most common methodologies for processing samples. The media used for lung tissue culture and CFU count varied widely, with Mueller-Hinton (I and II) as the preferred media (47.6% of the studies) followed by Luria-Bertani (LB) agar (14.3%). Agar plates were usually cultured at 37°C, although several studies reported an incubation temperature of 35°C for *P. aeruginosa.* It is important to optimize the culture conditions for inoculation and bacterial recovery from the lung. Different sample processing techniques and media may have an impact on the study results, and it is recommended that all study samples are processed using the same methodology. Working with blinded samples is strongly encouraged, as it may reduce the potential for bias and increase the robustness of the study (Ioannidis, 2012; Bespalov et al., 2020).

Monoparametric models employing a single indicator of antibiotic efficacy represented 39.6% of the studies reviewed. Lung bacterial burden was the most common study readout (Table 4). While some studies reported the data as CFU/lung, others reported the data as CFU/ml of homogenized lung. CFU/ml requires additional data (lung weight) for normalization and comparison of results between studies, but lung weight was not generally reported. Regardless of the reporting units, the reduction of bacterial burden at the site of infection provides a relatively reproducible measure of antibiotic action that has been shown to forecast drug efficacy in patients (Zak and O’Reilly, 1991; Andes and Lepak, 2017). Bacterial burden is generally the preferred endpoint because it is a direct measurement of the drug’s ability to kill or halt growth of the infecting pathogen. However, mortality can also be a useful endpoint and, in our review, survival of the mice was the second most common readout. The relationship between bacterial burden and survival has been previously noted, and the magnitude of drug exposure required for bacteriological cure and survival has been shown in some studies to be similar (Craig and Dalhoff, 1998; Andes and Craig, 2002). However, it should be noted that survival studies which monitor the animal’s health for prolonged periods of time after the end of therapy may allow organisms that have not been eradicated to regrow and cause mortality, especially in neutropenic animals. This could substantially impact the relationship between bacterial counts and survival (Andes and Craig, 2002). In addition to survival and bacterial burden in the lungs, other outcomes in the studies reviewed included lung histopathology, bacterial load in other tissues (e.g., blood, liver or spleen), assessment of immune responses (e.g., cytokines) in BAL, serum, or lung homogenate, clinical scoring based on animal observation, body temperature, protein expression, and pulmonary endothelial permeability.

## Conclusion

Standardization of the murine pneumonia model would allow better translation to the clinic and reduce animal use by providing more useful and relevant data. This literature review revealed marked differences in methodology for the murine pneumonia model used to test efficacy of small molecule antibiotics. Several parameters were relatively consistent across most of the models reviewed. These included animal sex, stage of bacterial growth for the inoculum, the starting point for treatment and the primary study outcome. Other variables differed widely, such as the immune status and age of the mice, the infection route and sample processing methodologies. Some variables, such as immune status and infectious dose, are expected to impact the study outcome more than others and can affect the PK/PD magnitude measured for a given endpoint. Although there is little direct evidence to indicate what effect, if any, a particular variable has on the outcome of a study, the myriad combinations of variables that any particular investigator uses is likely to impact the observed results. This can complicate preclinical-to-clinical translation, makes it difficult to compare drugs and/or bacterial isolates tested in different laboratories, and highlights the need for development of a standardized murine pneumonia model that has been benchmarked appropriately. Overall, standardization of animal infection models is expected to strengthen the reproducibility and comparability of data generated during the evaluation of novel antibiotics. Furthermore, the combination of standardized protocols with quality controls, such as bacterial reference strains and benchmark control compounds with specified potency, should increase the robustness of preclinical data and improve our ability to translate from animals to humans.

## Author contributions

RA: data curation, analysis, and writing—original draft. All authors: conceptualization, manuscript review and editing, and read and approved the final manuscript.
