# Supplementary material for: Variability of murine bacterial pneumonia models used to evaluate antimicrobial agents
Source: Front Microbiol. 2022 Sep 8;13:988728. doi: 10.3389/fmicb.2022.988728 (PMC9493352; doi:10.3389/fmicb.2022.988728)
Supplement: Supplementary file 1 [file Data_Sheet_1.docx]

**Variability of murine bacterial pneumonia models used to evaluate antimicrobial agents**

Rakel Arrazuria, Bernhard Kerscher, Karen E. Huber^1^, Jennifer L. Hoover, Carina Vingsbo Lundberg, Jon Ulf Hansen, Sylvie Sordello, Stephane Renard, Vincent Aranzana-Climent, Diarmaid Hughes, Philip Gribbon, Lena E. Friberg, Isabelle Bekeredjian-Ding.

**Supplementary Table 1**. Mesh and Free terms employed for study PubMed search on 10^th^ March, 2021.

| Mesh and Free terms |
| --- |
| ("Mice" [Mesh] OR "Models, Animal"[Mesh] OR "Disease Models, Animal"[Mesh] OR "mice" [Tiab] OR "mus" [Tiab] OR "mouse" [Tiab] OR "murine" [Tiab] OR "woodmouse" [Tiab] OR "murinae" [Tiab] OR "muridae" [Tiab] OR "Mice lung infection" [tiab] OR "Mouse lung infection" [tiab] OR "preclinical murine model"[tiab]) AND ("Pseudomonas"[Mesh] OR "Acinetobacter"[Mesh] OR "Klebsiella"[Mesh] OR "Gram-Negative Aerobic Bacteria"[Mesh]) AND ("Pneumonia"[Mesh] OR "Lung/microbiology"[Mesh] OR "Lung/pharmacology"[Mesh] OR "Healthcare-Associated Pneumonia/drug therapy"[Mesh] OR "Healthcare-Associated Pneumonia/microbiology"[Mesh] OR "Healthcare-Associated Pneumonia/therapy"[Mesh] OR "hospital acquired pneumonia" [tiab] OR "ventilator-associated pneumonia" [tiab]) AND ("Pneumonia, Bacterial/drug therapy"[Mesh] OR "Pneumonia, Bacterial/microbiology"[Mesh] OR "Anti-Bacterial Agents/administration and dosage"[Mesh] OR "Anti-Bacterial Agents/drug therapy"[Mesh] OR "Anti-Bacterial Agents/microbiology"[Mesh] OR "Anti-Bacterial Agents/pharmacokinetics"[Mesh] OR "Anti-Bacterial Agents/pharmacology"[Mesh] OR "Anti-Bacterial Agents/therapeutic use"[Mesh] OR "Microbial Viability/drug effects"[Mesh] OR "Drug Tolerance"[Mesh] OR "Drug Synergism"[Mesh] OR "Drug Therapy, Combination"[Mesh] OR "PK/PD"[tiab]) |

**Supplementary Table 2.** Reviewed studies dataset.

| **Author** | **Journal** | **Year** | **Bacteria** | **DOI** |
| --- | --- | --- | --- | --- |
| Johnson A *et al.* | Antimicrob Agents Chemother | 2020 | *K. pneumoniae* | doi: 10.1128/AAC.00180-20 |
| Tan S *et al.* | Sci Rep. | 2020 | *K. pneumoniae* | doi: 10.1038/s41598-020-76895-5 |
| Ma XL *et al.* | Sci Rep. | 2020 | *A. baumannii* | doi: 10.1038/s41598-020-74503-0 |
| Ku NS *et al.* | Sci Rep. | 2019 | *A. baumannii* | doi: 10.1038/s41598-019-53714-0 |
| Ren H *et al.* | J Infect Dis | 2019 | *P. aeruginosa* | doi: 10.1093/infdis/jiz341 |
| Zhao M *et al.* | Antimicrob Agents Chemother | 2019 | Combination | doi: 10.1128/AAC.01131-19 |
| Nakamura R *et al.* | Antimicrob Agents Chemother | 2019 | Combination | doi: 10.1128/AAC.02031-18 |
| Kirby BD *et al.* | Antimicrob Agents Chemother | 2019 | *P. aeruginosa* | doi: 10.1128/AAC.02341-18 |
| Sanderink D *et al.* | Future Microbiol. | 2019 | *A. baumannii* | doi: 10.2217/fmb-2019-0022 |
| Avery LM *et al.* | Antimicrob Agents Chemother | 2018 | *A. baumannii* | doi: 10.1128/AAC.00948-18 |
| Lou W *et al.* | Acta Biomater | 2018 | *K. pneumoniae* | doi: 10.1016/j.actbio.2018.07.038 |
| Geller BL *et al.* | J Antimicrob Chemother | 2018 | *K. pneumoniae* | doi: 10.1093/jac/dky058 |
| de Paula TP *et al.* | Antimicrob Agents Chemother | 2018 | *K. pneumoniae* | doi: 10.1128/AAC.00764-17 |
| Lin YW *et al.* | Antimicrob Agents Chemother | 2018 | Combination | doi: 10.1128/AAC.01790-17 |
| Monogue ML *et al.* | Pharmacology | 2018 | *A. baumannii* | doi: 10.1159/000486445 |
| Chen C *et al.* | Clin Microbiol Infect | 2018 | *P. aeruginosa* | doi: 10.1016/j.cmi.2017.08.029 |
| Lin YW et al. | Antimicrob Agents Chemother | 2017 | *P. aeruginosa* | doi: 10.1128/AAC.00211-17 |
| Kaku N *et al.* | Antimicrob Agents Chemother | 2017 | *K. pneumoniae* | doi: 10.1128/AAC.00828-17 |
| Zhou J *et al.* | Antimicrob Agents Chemother | 2017 | *A. baumannii* | doi: 10.1128/AAC.02371-16 |
| Zhou YF *et al.* | Antimicrob Agents Chemother | 2017 | *K. pneumoniae* | doi: 10.1128/AAC.02691-16 |
| Kaku N *et al.* | Antimicrob Agents Chemother | 2017 | *P. aeruginosa* | doi: 10.1093/jac/dkw517 |
| Lin YW *et al.* | Antimicrob Agents Chemother | 2017 | *P. aeruginosa* | doi: 10.1128/AAC.02025-16 |
| Sakoulas G *et al.* | Antimicrob Agents Chemother | 2017 | *A. baumannii* | doi: 10.1128/AAC.01745-16 |
| Li Y *et al.* | Cell Physiol Biochem | 2017 | *P. aeruginosa* | doi: 10.1159/000479411 |
| Oshima K *et al.* | Antimicrob Agents Chemother | 2016 | *P. aeruginosa* | doi: 10.1128/AAC.02056-16 |
| Thabit AK *et al.* | Int J Antimicrob Agents | 2016 | *K. pneumoniae* | doi: 10.1016/j.ijantimicag.2016.08.012 |
| Li Y *et al.* | Pharm Dev Technol | 2017 | *P. aeruginosa* | doi: 10.1080/10837450.2016.1228666 |
| McCaughey LC *et al.* | Sci Rep. | 2016 | *P. aeruginosa* | doi: 10.1038/srep30201 |
| Cigana C *et al.* | Antimicrob Agents Chemother | 2016 | *P. aeruginosa* | doi: 10.1128/AAC.00390-16 |
| Lepak AJ *et al.* | Antimicrob Agents Chemother | 2016 | *K. pneumoniae* | doi: 10.1128/AAC.00647-16 |
| Parra Millán R *et al.* | Antimicrob Agents Chemother | 2016 | *A. baumannii* | doi: 10.1128/AAC.02708-15 |
| Yang YS *et al.* | Antimicrob Agents Chemother | 2016 | *A. baumannii* | doi: 10.1128/AAC.02994-15 |
| Mardirossian *et al.* | Amino Acids. | 2016 | *P. aeruginosa* | doi: 10.1007/s00726-016-2266-4 |
| Brunetti J *et al.* | Sci Rep. | 2016 | *P. aeruginosa* | doi: 10.1038/srep26077 |
| Berkhout J *et al.* | Antimicrob Agents Chemother | 2015 | *P. aeruginosa* | doi: 10.1128/AAC.01269-15 |
| Cheah SE *et al.* | Antimicrob Agents Chemother | 2015 | Combination | doi: 10.1093/jac/dkv267 |
| Bowers DR *et al.* | Antimicrob Agents Chemother | 2015 | *A. baumannii* | doi: 10.1128/AAC.04110-14 |
| Louie A *et al.* | J Infect Dis | 2015 | *P. aeruginosa* | doi: 10.1093/infdis/jiu603 |
| Harada Y *et al.* | Clin Microbiol Infect | 2014 | *K. pneumoniae* | doi: 10.1111/1469-0691.12677 |
| Yokoyama Y *et al.* | Int J Antimicrob Agents | 2014 | *A. baumannii* | doi: 10.1016/j.ijantimicag.2014.02.012 |
| Hengzhuang W *et al.* | Methods Mol Biol | 2014 | *P. aeruginosa* | doi: 10.1007/978-1-4939-0467-9_17 |
| He J *et al.* | Int J Antimicrob Agents | 2013 | *P. aeruginosa* | doi: 10.1016/j.ijantimicag.2013.07.009 |
| Yamada K *et al.* | Int J Antimicrob Agents | 2013 | *P. aeruginosa* | doi: 10.1016/j.ijantimicag.2013.05.016 |
| Yamada K *et al.* | Antimicrob Agents Chemother | 2013 | *A. baumannii* | doi: 10.1128/AAC.00457-13 |
| Louie A *et al.* | Antimicrob Agents Chemother | 2013 | *P. aeruginosa* | doi: 10.1128/AAC.02624-12 |
| Hirsch EB *et al.* | J Infect Dis | 2013 | *K. pneumoniae* | doi: 10.1093/infdis/jis766 |
| Jacqueline C *et al.* | J Antimicrob Chemother | 2013 | *P. aeruginosa* | doi: 10.1093/jac/dks343 |
| Hengzhuang W *et al.* | Antimicrob Agents Chemother | 2012 | *P. aeruginosa* | doi: 10.1128/AAC.06486-11 |
| Yamada K *et al.* | J Infect Chemother | 2012 | *P. aeruginosa* | doi: 10.1007/s10156-011-0359-2 |
| Docobo-Pérez F *et al.* | Int J Antimicrob Agents | 2012 | *K. pneumoniae* | doi: 10.1016/j.ijantimicag.2011.10.012 |
| Tang HJ *et al.* | Int J Infect Dis | 2012 | *A. baumannii* | doi: 10.1016/j.ijid.2011.09.015 |
| López-Rojas R *et al.* | Int J Antimicrob Agents | 2011 | *A. baumannii* | doi: 10.1016/j.ijantimicag.2011.06.006 |
| Pachón-Ibáñez ME *et al.* | Eur J Clin Microbiol Infect Dis | 2011 | *A. baumannii* | doi: 10.1007/s10096-011-1173-6 |
